# Microelectromechanical reprogrammable logic device

**DOI:** 10.1038/ncomms11137

**Published:** 2016-03-29

**Authors:** M. A. A. Hafiz, L. Kosuru, M. I. Younis

**Affiliations:** 1 Physical Sciences and Engineering Division, King Abdullah University of Science and Technology, Thuwal 23955-6900, Saudi Arabia

## Abstract

In modern computing, the Boolean logic operations are set by interconnect schemes between the transistors. As the miniaturization in the component level to enhance the computational power is rapidly approaching physical limits, alternative computing methods are vigorously pursued. One of the desired aspects in the future computing approaches is the provision for hardware reconfigurability at run time to allow enhanced functionality. Here we demonstrate a reprogrammable logic device based on the electrothermal frequency modulation scheme of a single microelectromechanical resonator, capable of performing all the fundamental 2-bit logic functions as well as *n*-bit logic operations. Logic functions are performed by actively tuning the linear resonance frequency of the resonator operated at room temperature and under modest vacuum conditions, reprogrammable by the a.c.-driving frequency. The device is fabricated using complementary metal oxide semiconductor compatible mass fabrication process, suitable for on-chip integration, and promises an alternative electromechanical computing scheme.

## Abstract (web summary)

Microelectromechanical resonators behave as logic gates thanks to the tunability of their resonance frequencies. Here, the authors combine the mechanical response to electrothermal frequency modulation to demonstrate 2- and 3-bit reprogrammable logic gates at room temperature and under modest vacuum.

## Graphical abstract

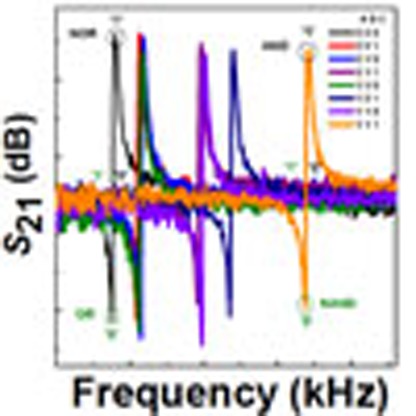

The quest for mechanical computation is a century old and can be traced back to at least 1822 when Babbage presented his concept of difference engine^1^. Although the interest remained within the research community, the subsequent development in the fields of electronic transistor^2^ and magnetic storage^3^,^4^ outperformed the mechanical approach in computation both in terms of speed of operation and data density. However, recent advancements in micro-/nano-fabrication and measurement techniques have renewed the interest in the field of mechanical computation in the last decade^5^,^6^,^7^,^8^,^9^,^10^,^11^,^12^,^13^,^14^,^15^,^16^,^17^,^18^,^19^,^20^,^21^.

The key to any computing machine are logic elements. The first demonstrated dynamic mechanical XOR logic gate was based on a piezoelectric nanoelectromechanical system (NEMS) structure where the presence (absence) of high-amplitude vibration in the linear regime denotes a logical high (low) state^7^. Later, OR/NOR and AND/NAND logic gates have been demonstrated utilizing the bistability of a nonlinearly resonating NEMS resonator mediated by the noise floor^12^. A universal logic device capable of performing AND, OR and XOR logic gates as well as multibit logic circuits has been implemented by parametrically exciting a single electromechanical resonator^15^. Same research group also demonstrated XOR and OR logic gates in an electromechanical membrane resonator under high vacuum and at room temperature condition^16^. On the basis of feedback control, a memory and OR logic operation have been demonstrated on a single microelectromechanical system (MEMS) resonator working in the nonlinear regime^20^. Recently, an unconventional and reversible logic gate (Fredkin gate) has been presented based on four coupled linearly resonating NEMS resonators^21^ where AND, OR, NOT and FANOUT gate operations have been demonstrated. Note that room temperature and atmospheric operations are desirable prerequisites for any practical device implementation.

Here we demonstrate a reprogrammable logic device, capable of performing 2-bit AND, NAND, OR, NOR, XOR, XNOR and NOT logic operations using a single microelectromechanical resonator operating in the linear regime. The logic operations are performed by electrothermal modulation of the linear resonance frequency of the resonator, where two separate d.c. voltage sources represent logic inputs. The device can be programmed to perform any of these logic operations by simply tuning the a.c.-driving frequency. Also, we use this scheme of electrothermal frequency tuning to demonstrate 3-bit AND, NAND, OR and NOR logic gates on a single MEMS resonator. This can be extended to *n*-bit logic operations by adding a single d.c. voltage source per bit. This device works under room temperature and modest vacuum conditions and is fabricated using standard complementary metal oxide semiconductor-based fabrication techniques suitable for mass fabrication and on-chip integrated system development.

## Results

### Device fabrication and experimental set-up

The resonator is fabricated on a highly conductive Si device layer of silicon on insulator wafer by a two-mask process using standard photolithography, electron beam evaporation for metal layer deposition for actuating pad, deep reactive ion etch for silicon device layer etching and vapour hydrofluoric acid etch to remove the oxide layer underneath the resonating structure. It consists of a clamped–clamped arch-shaped microbeam with two adjacent electrodes to electrostatically induce the vibration and detect the generated a.c. output current due to the in-plane motion of the microbeam. The dimensions of the curved beam are 500 μm in length, 3 μm in width and 30 μm in thickness. The gap between the actuating electrode and the resonating beam is 8 μm at the fixed anchors and 11 μm at the midpoint of the microbeam due to its 3-μm initial curvature.

**Figure 1:**
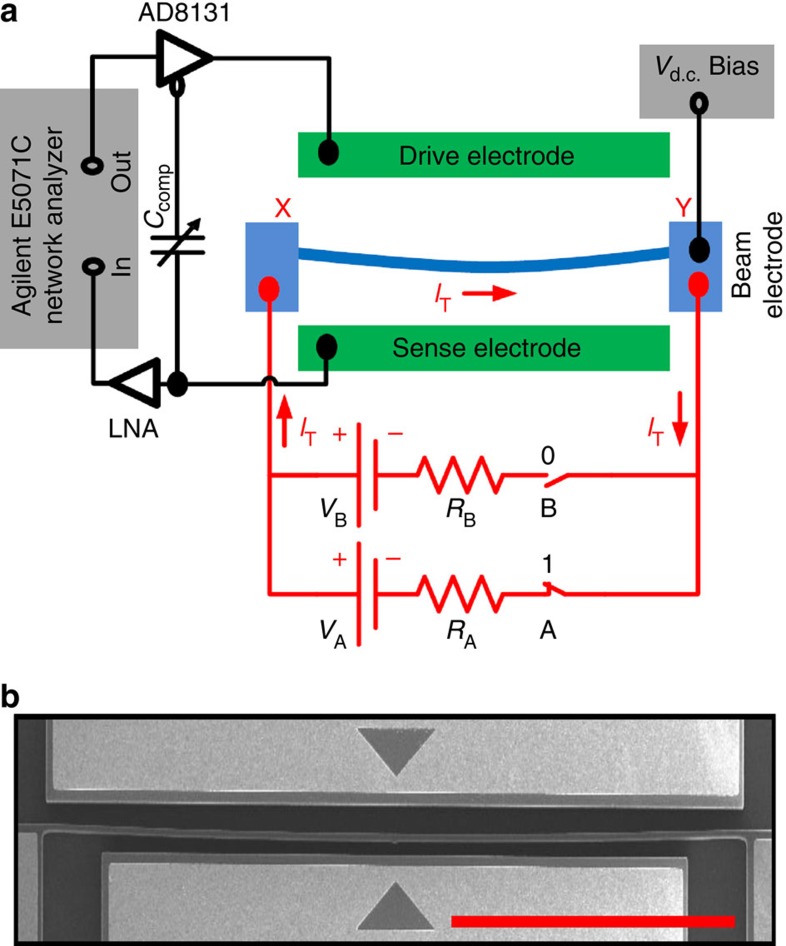
Clamped–clamped arch resonator. (**a**) Schematic of the arch beam resonator and the two-port electrical transmission measurement configuration together with a parasitic current compensation circuitry using single-to-differential driver (AD8131) and a variable compensation capacitor, *C*_comp_. The drive electrode is provided with an a.c. signal from one of the outputs from AD8131 and the beam electrode is biased with a d.c. voltage source. The output current induced at the sense-electrode is coupled with the compensation capacitor and followed by a low-noise amplifier (LNA) whose output is coupled to the network analyser input port. Two voltage sources, *V*_A_ and *V*_B_ and switches, A and B are connected in parallel across the beam to perform logic operations by electrothermal tuning of the resonance frequency. The arrow in the red represents the current flowing through the beam, responsible for electrothermal frequency modulation. (**b**) An SEM image of the microbeam resonator. Scale bar, 200 μm.

Figure 1a shows the schematic of the arch microbeam and the two-port electrical transmission measurement configuration for electrostatic actuation and sensing that includes the parasitic current compensation circuit for enhanced transmission signal measurements^22^. The drive electrode is provided with an a.c. actuation signal from one of the outputs of a single-to-differential driver (AD8131), and the beam electrode is biased with a d.c. voltage source. The output current induced at the sense electrode is coupled with the variable compensation capacitor, *C*_comp_, and followed by a low-noise amplifier whose output is coupled to the network analyser input port. Two logic inputs are provided with two d.c. voltage sources, *V*_A_ and *V*_B_, connected in parallel across the microbeam with series resistors, *R*_A_ and *R*_B_, and switches, A and B, respectively. The electrical wiring scheme for the logic inputs is depicted in red to differentiate it from the rest of the electrical connections. The binary logic input 1(0) is represented by connecting (disconnecting) *V*_A_ and *V*_B_ from the electrical network by the two switches, A and B, respectively. Hereafter, switch ON (OFF) condition for switches A and B corresponds to the binary logic input 1(0). The sensing electrode is used to obtain the logic output, where a relative high (low) *S*_21_ transmission signal corresponds to the logic output 1(0). Figure 1b shows an SEM image of the arch microbeam resonator.

### Electrothermal frequency modulation

**Figure 2:**
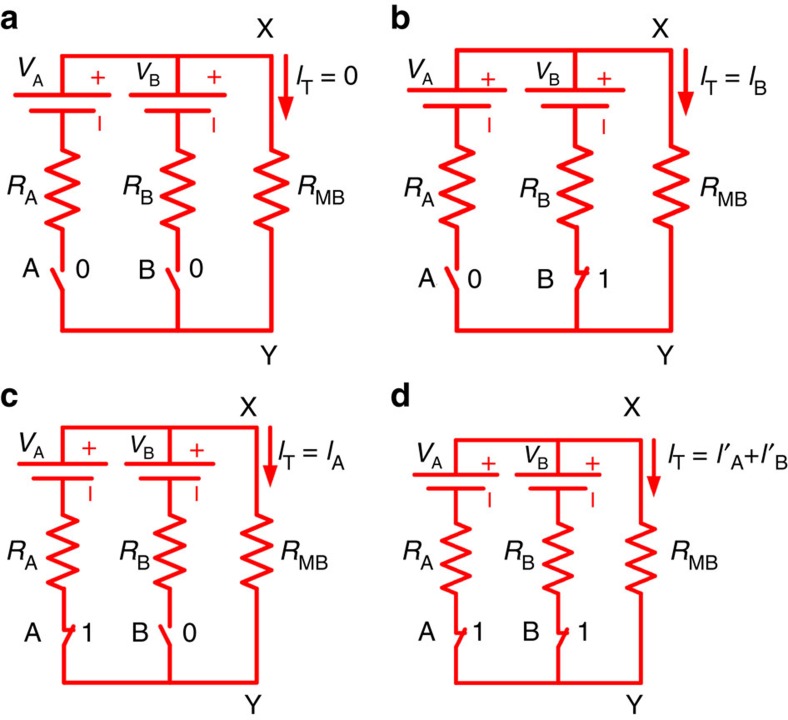
Electrical circuit configuration of the logic input conditions. (**a**) The electrical circuit represents the (0,0) logic input condition where the total current *I*_T_ through the beam *R*_MB_ is zero. (**b**) The circuit represents the (0,1) logic input condition corresponds to switch A, OFF and switch B, ON where the total current *I*_T_ flowing through the beam *R*_MB_ is *I*_B_. (**c**) The circuit represents the (1,0) logic input condition corresponds to switch A, ON and switch B, OFF where the total current *I*_T_ flowing through the beam *R*_MB_ is *I*_A_. (**d**) The circuit represents the (1,1) logic input condition corresponds to switch A, ON and switch B, ON where the total current *I*_T_ flowing through the beam *R*_MB_ is *I*′_A_+*I*′_B_.

**Figure 3:**
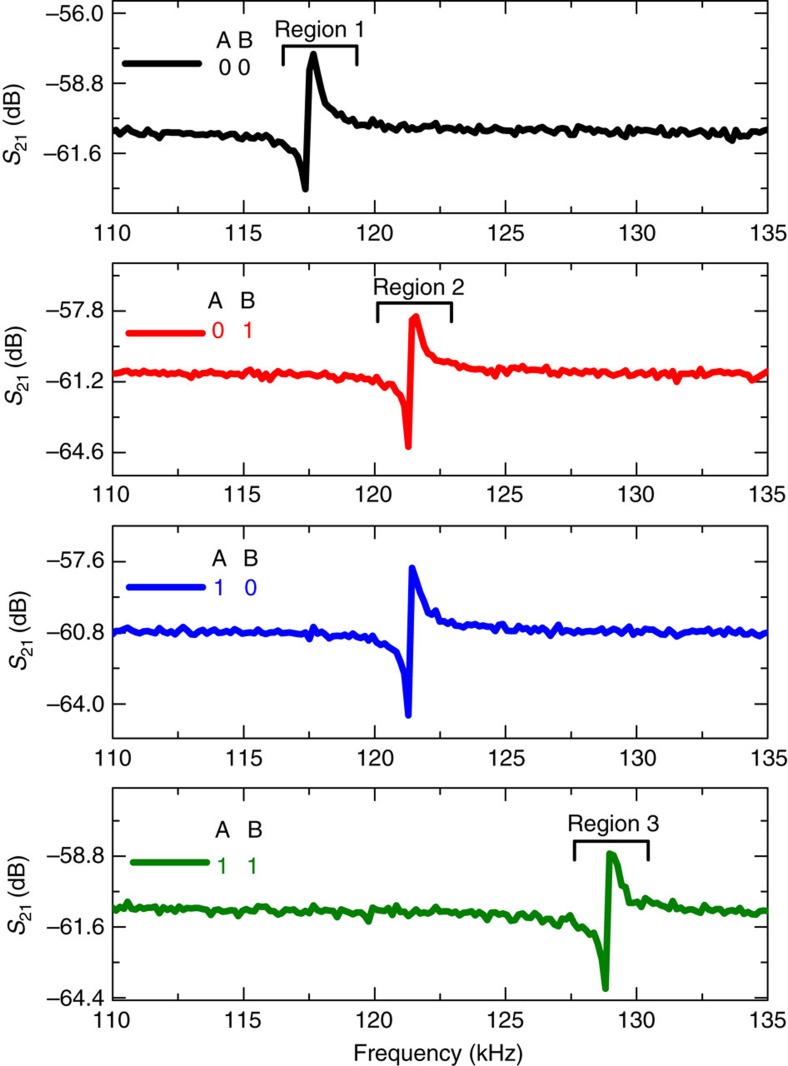
Electrothermal frequency modulation. Frequency responses of the resonator for different logic input conditions, (0,0), (0,1), (1,0) and (1,1), shown in black, red, blue and green, respectively.

Electrothermal frequency modulation has an essential role in the execution of the logic functions in this architecture. Figure 2 shows four different electrical circuit configurations between nodes X and Y, shown in Fig. 1a. All the four logic input conditions, (0,0), (0,1), (1,0) and (1,1) are shown in Fig. 2a–d, respectively. For the case of (0,0) logic input condition, the total current flowing through the microbeam is *I*_T_=0 as depicted in the electrical circuit in Fig. 2a. In this case, the resonator exhibits series resonance peak and parallel resonance dip (anti-resonance) at 117.663 and 117.361 kHz, respectively, with an a.c. actuation voltage of 2 dBm (0.28 *V*_rms_) and *V*_d.c._ of 45 V at 1 torr pressure and at room temperature (see Supplementary Note 1 and Supplementary Fig. 1a,b). The corresponding frequency response is plotted in black in Fig. 3. Note that due to over compensation of the feed through by the parallel variable compensation capacitance, *C*_comp_, the parallel resonance appears earlier than the series resonance^22^. However, this does not put any limitation on the successful logic operation by the device. Moreover, we use both the series and parallel resonances for implementing the logic gates. For logic input (0,1) or (1,0) conditions, either *V*_B_ or *V*_A_ is connected to the microbeam as depicted in the electrical circuits shown in Fig. 2b,c, respectively. Hence, the total current that flows through the microbeam is either *I*_T_=*I*_B_ or *I*_T_=*I*_A_. We chose *V*_A_=0.4 V, *V*_B_=0.7 V, and *R*_A_=*R*_B_=50 Ω so that it satisfies the condition of the same current amount at each case; *I*_A_=*I*_B_. Note that we measured the microbeam resistance *R*_MB_=114 Ω. The electrical current flowing through the microbeam generates heat and causes thermal expansion, which induces compressive axial force. This compressive force causes an increase in the microbeam curvature^23^,^24^,^25^ and increases its stiffness. Hence, the series resonance frequency increases to 121.431 kHz for either (0,1) or (1,0) logic input conditions. The frequency responses due to the logic input (0,1) and (1,0) conditions are plotted as red and blue, respectively, in Fig. 3. For logic input condition (1,1), both the voltage sources *V*_A_ and *V*_B_ are connected to the microbeam as depicted in the electrical circuit shown in Fig. 2d. The total current generated in this case is *I*_T_=*I*′_A_+*I*′_B_>*I*_A_ or *I*_B_. Hence, the series resonance frequency further increases to 128.969 kHz as depicted in green in Fig. 3. Thus, one can modulate the resonance frequencies (series and parallel) of the microbeam through the electrothermal effect by controlling the amount of current flow in the microbeam. Towards this, we build different logic gates by properly choosing the a.c.-driving frequency. We identify three regions in the frequency response plot of Fig. 3 to build all the six logic gates. Region I corresponds to frequency of operation for logic gates OR/NOR, region II corresponds to logic gates XOR/XNOR and finally, region III corresponds to logic gates AND/NAND. NOT logic operation can be built on any of these frequencies by proper conditioning of one of the inputs. The detail execution of the logic gates will be discussed in the following sections.

### NOR/OR

**Figure 4:**
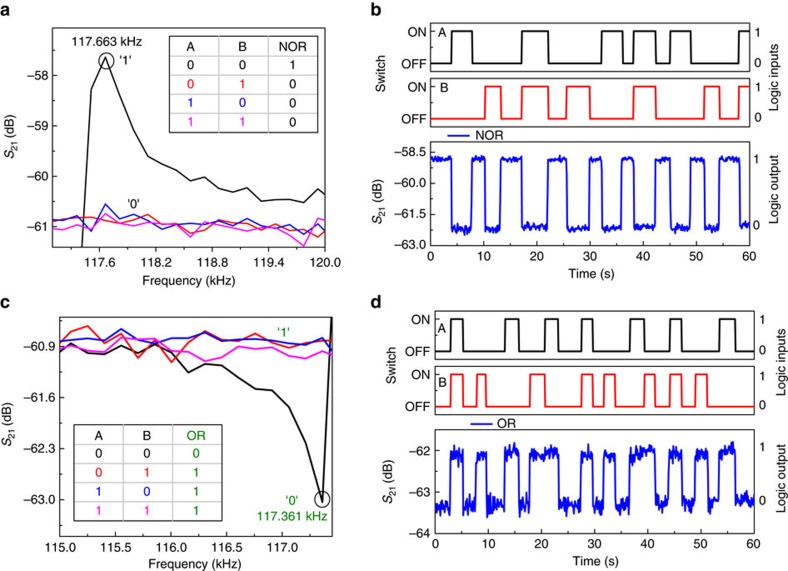
Demonstration of 2-bit NOR and OR logic gates. (**a**) Frequency responses of the resonator for different logic input conditions where (0,0) logic input condition, shown in black has high *S*_21_ transmission signal at 117.663 kHz and others have low *S*_21_ transmission signal represented by 1 and 0, respectively. Truth table of NOR logic output is shown in the inset. (**b**) Demonstration of NOR logic operation when the frequency of the a.c. input signal is chosen as 117.663 kHz. Two input signals A and B are shown in black and red, respectively, where the switch OFF/ON corresponds to 0/1 logic input conditions. The *S*_21_ transmission signal in blue corresponds to the logic output and fulfills the NOR truth table. (**c**) Frequency responses of the resonator for different logic input conditions, where (0,0) logic input condition shown in black has low *S*_21_ transmission signal at 117.361 kHz and others have high *S*_21_ transmission signal, represented by 0 and 1, respectively. Truth table for OR logic output shown in the inset. (**d**) Demonstration of OR logic operation when the a.c. input signal frequency is chosen as 117.361 kHz. Two input signals, A and B are shown in black and red, respectively, and the switch OFF/ON corresponds to 0/1 logic input conditions. The *S*_21_ transmission signal in blue corresponds to the logic output that fulfills the OR truth table.

The frequency responses of the resonator for different logic input conditions are shown in Fig. 4a, which lies in the region I of Fig. 3. To demonstrate NOR gate operation, the frequency of 117.663 kHz is chosen as it shows high *S*_21_ transmission signal denoted as the logic output 1 (in black) for (0,0) logic input condition only. The resonator is tuned away from its series resonance frequency of 117.663 kHz by other logic input conditions, (0,1), (1,0) and (1,1), respectively. Hence, shows low *S*_21_ transmission signal denoted as logic output 0 (in black) at the frequency of 117.663 kHz. The NOR gate truth table is shown in the inset of Fig. 4a. The time response of the resonator showing binary inputs A and B and the corresponding logic output is depicted in Fig. 4b. It clearly shows NOR logic operation as the output is 1 (high) only when both the inputs A and B are 0 (switch OFF), and the output is 0 (low) for all the other conditions, (0,1), (1,0) and (1,1).

To demonstrate OR logic gate, we exploit the parallel resonance dip at 117.361 kHz, shown in black circle in Fig. 4c. Here the low level of *S*_21_ transmission signal is considered as the logic output 0 (in green), and otherwise as the logic output 1 (in green). The OR gate truth table is shown in the inset of Fig. 4c. Figure 4d shows the time response of the resonator output for OR logic gate operation with the corresponding binary inputs A and B. It clearly shows OR logic operation as the logic output is 0 (low) when both the inputs A and B are 0, and logic output is 1 (high) for all the other conditions.

### NOT

**Figure 5:**
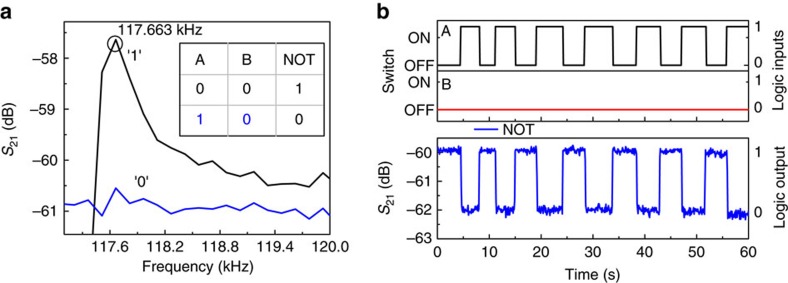
Demonstration of NOT gate. (**a**) Frequency responses of the resonator for different logic input conditions, where (0,0) logic input condition shown in black has high *S*_21_ transmission signal at 117.663 kHz and others have low *S*_21_ transmission signal represented by 1 and 0, respectively. Truth table of NOT logic gate is shown in the inset. (**b**) Demonstration of NOT logic operation when the frequency of the a.c. input signal is chosen as 117.663 kHz. Two input signals, A and B are shown in black and red, respectively, where the switch OFF/ON corresponds to 0/1 logic input conditions. *S*_21_ transmission signal in blue corresponds to the logic output and fulfills the NOT truth table.

To perform NOT operation on the input A, the a.c.-driving frequency is set to be at 117.663 kHz and the input B is set to 0 (switch OFF). For this set condition, a high *S*_21_ transmission signal (logic output 1) is achieved for the logic input A set at 0 (switch OFF) and vice versa as shown in Fig. 5a. We note that NOT operation can also be built on input B by properly setting input A (switch OFF/ON) and a.c.-driving frequency. The time response for the NOT operation is shown in Fig. 5b. It is evident from the output signal that when the input A is 0, the output is 1 and vice versa.

### XOR/XNOR

**Figure 6:**
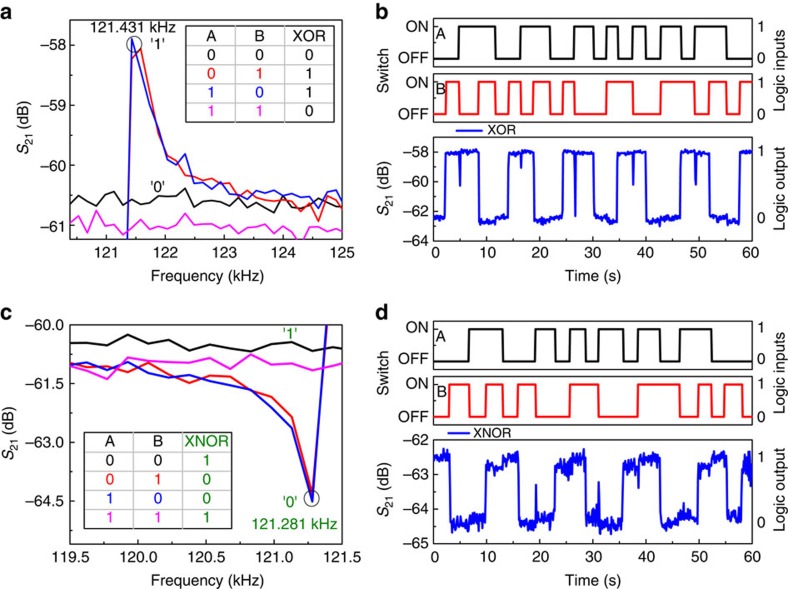
Demonstration of 2-bit XOR and XNOR logic gates. (**a**) Frequency responses of the resonator for different logic input conditions, where (0,1) and (1,0) logic input condition shown in red and blue has high *S*_21_ transmission signal at 121.43 kHz and others have low *S*_21_ transmission signal represented by 1 and 0, respectively. Truth table of XOR logic gate is shown in the inset. (**b**) Demonstration of XOR logic operation when the operation frequency is chosen as 121.43 kHz. Two input signals, A and B are shown in black and red, respectively, where the switch OFF/ON corresponds to 0/1 logic input conditions. *S*_21_ transmission signal in blue corresponds to the logic output that fulfills the XOR truth table. (**c**) Frequency responses of the resonator for different logic input conditions, where (0,0) and (1,1) logic input conditions shown in red and blue, respectively, has low *S*_21_ transmission signal at 121.281 kHz and others have high *S*_21_ transmission signal represented by 0 and 1, respectively. Truth table of XNOR logic output is shown in the inset. (**d**) Demonstration of XNOR logic operation when the operating frequency is fixed at 121.281 kHz. Two input signals, A and B are shown in black and red, respectively, where the switch OFF/ON corresponds to 0/1 logic input conditions. *S*_21_ transmission signal in blue corresponds to the logic output and fulfills the XNOR truth table.

Frequency responses of the resonator for different logic input conditions are shown in Fig. 6a, which lies in the region II of Fig. 3. To implement XOR gate, the frequency of operation is chosen as 121.431 kHz, shown in black circle in Fig. 6a. At this operating frequency, it shows low *S*_21_ transmission signal denoted as the logic output 0 (in black) for the logic input conditions (0,0) and (1,1). For other logic input conditions, (0,1) and (1,0), it shows high *S*_21_ transmission signal denoted as the logic output 1 (in black). The truth table for XOR logic gate is shown in the inset of Fig. 6a. Figure 6b shows the time response of the resonator output for XOR logic gate operation with the corresponding binary inputs A and B. It clearly shows XOR logic gate operation as the logic output is 1 (high) when the inputs A and B are complementary to each other. On the other hand, the logic output is 0 (low) for the same logic input conditions, (0,0) and (1,1).

To demonstrate XNOR logic gate, we exploit the parallel resonance dip at 121.281 kHz, shown in black circle in Fig. 6c. Here the low level of *S*_21_ transmission signal is considered as the logic output 0 (in green), and otherwise as the logic output 1 (in green). XNOR truth table is shown in the inset of Fig. 6c. Figure 6d shows the time response of XNOR logic gate output and the corresponding binary logic inputs A and B. It clearly shows XNOR logic gate operation as the logic output is 1 (high) when both the inputs A and B are same, (0,0) and (1,1), and otherwise the logic output is 0 (low). Note that occasional spikes observed in the *S*_21_ transmission signal (in blue) in Fig. 6b,d are due to the switching between (0,1) and (1,0) logic input conditions. However, the resonator still performs the desired logic operations successfully.

### AND/NAND

**Figure 7:**
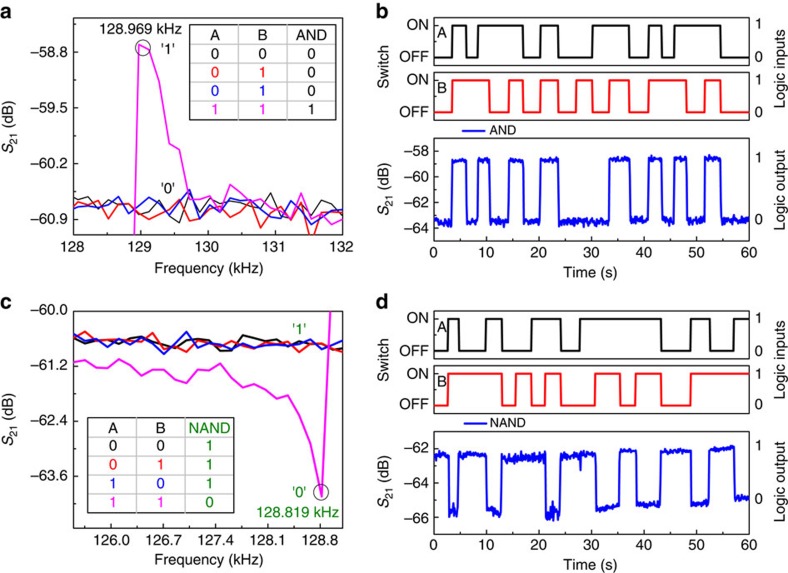
Demonstration of 2-bit AND and NAND logic gates. (**a**) Frequency responses of the resonator for different logic input conditions, where (1,1) logic input condition shown in magenta has high *S*_21_ transmission signal at 128.969 kHz and others have low signal represented by 1 and 0, respectively. Truth table of AND logic output is shown in the inset. (**b**) Demonstration of AND logic operation when the operation of frequency is chosen as 128.969 kHz. Two input signals, A and B are shown in black and red, respectively, where the switch OFF/ON corresponds to 0/1 logic input conditions. S_*2*1_ transmission signal in blue corresponds to the logic output and fulfills the AND truth table. (**c**) Frequency responses of the resonator for different logic input conditions, where (1,1) logic input condition has low *S*_21_ transmission signal at 128.819 kHz and others have high *S*_21_ transmission signal represented by 0 and 1, respectively. Truth table of NAND logic output is shown in the inset. (**d**) Demonstration of NAND logic operation when the operation of frequency is chosen as 128.819 kHz. Two input signals, A and B are shown in black and red, respectively, where the switch OFF/ON corresponds to 0/1 logic input conditions. *S*_21_ transmission signal in blue corresponds to the logic output and fulfills the NAND truth table.

Frequency responses of the resonator for different logic input conditions are shown in Fig. 7a, which falls in the region III of Fig. 3. To demonstrate AND gate operation, the frequency of 128.969 kHz is chosen, which is shown in black circle in Fig. 7a. When both the inputs A and B are 1 (switch ON), the high *S*_21_ transmission signal is observed at this operating frequency and denoted as the logic output 1 (in black). For other logic input conditions, (0,1), (1,0) and (0,0), it shows the low *S*_21_ transmission signal, which is denoted as the logic output 0 (in black). This is expressed in a truth table in the inset of Fig. 7a. The time response of the resonator for AND gate operation and the corresponding binary logic inputs A and B are shown in Fig. 7b. It clearly shows AND gate operation as the output is 1 (high) only when both the inputs A and B are 1, otherwise 0 (low).

To demonstrate NAND gate, the frequency of operation is chosen at 128.819 kHz, shown in black circle in Fig. 7c. Here the low level of *S*_21_ transmission signal of the parallel resonance dip is considered as the logic output 0 (in green), and otherwise as the logic output 1 (in green). NAND gate truth table is shown in the inset of Fig. 7c. Figure 7d shows the time response of NAND logic gate output and the corresponding binary logic inputs A and B. It shows NAND logic operation as the logic output is 0 (low) only when both the inputs A and B are 1 (switch ON).

### Three-bit logic gates

**Figure 8:**
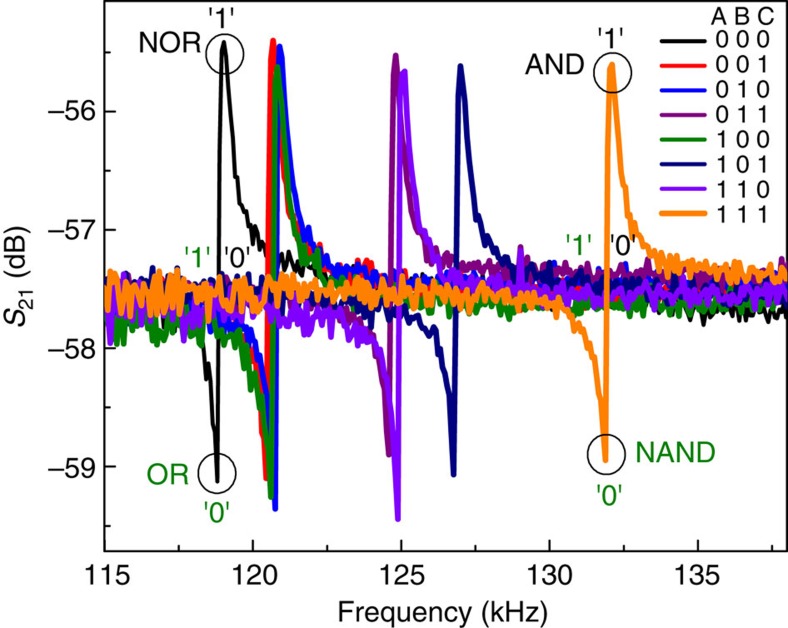
Realization of 3-bit logic gates. Frequency responses of the resonator for three different input logic conditions. NOR gate is realized by choosing the frequency of operation at 119.022 kHz, where (0,0,0) logic input condition has high *S*_21_ transmission signal and all the others have low *S*_21_ transmission signal. By choosing the corresponding anti resonance dip frequency, 3-bit OR gate can be realized. A 3-bit AND gate is realized by choosing the frequency of operation at 132.105 kHz, where (1,1,1) logic input condition has high *S*_21_ transmission signal and all others have low *S*_21_ transmission signal. By choosing the corresponding anti resonance dip frequency, a 3-bit NAND gate can be realized.

**Figure 9:**
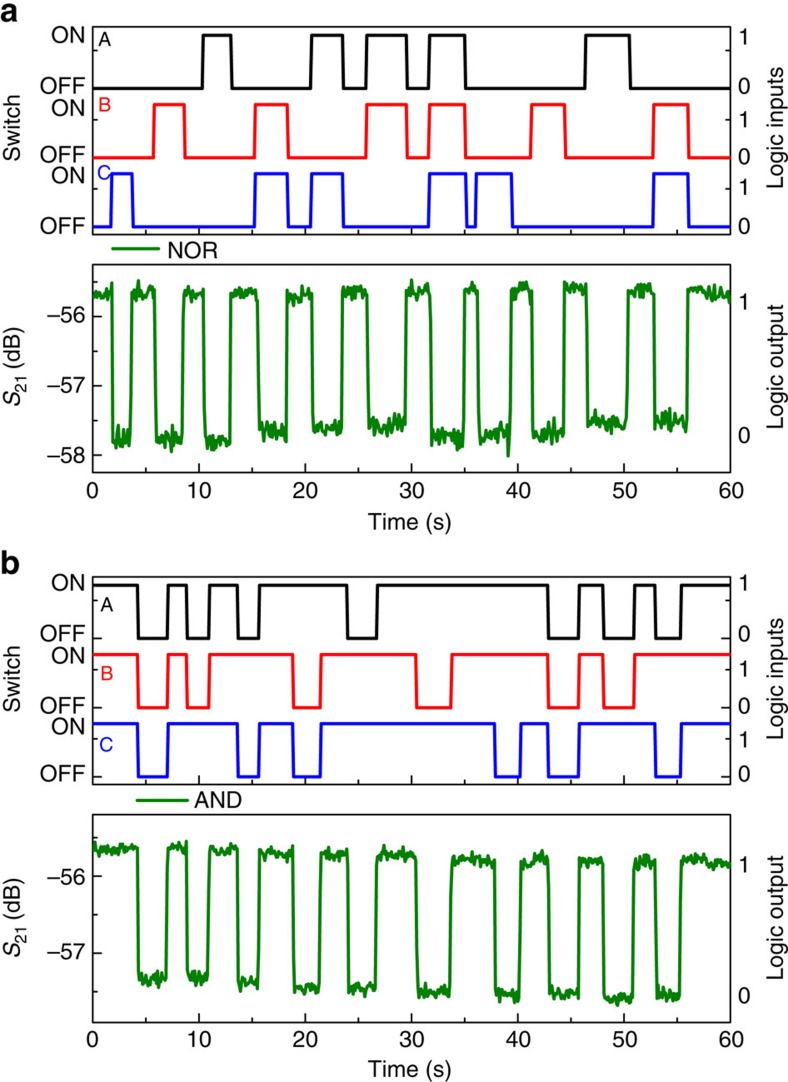
Demonstration of 3-bit logic gates. (**a**) Demonstration of 3-bit NOR logic operation when the operating frequency is chosen at 119.022 kHz. Three input signals, A, B, and C are shown in black, red, and blue, respectively, where the switch OFF/ON corresponds to 0/1 logic input conditions. *S*_21_ transmission signal in green corresponds to the logic output and fulfills the NOR truth table. (**b**) Demonstration of 3-bit AND logic operation when the operation frequency is chosen at 132.105 kHz. Three input signals, A, B, and C are shown in black, red, and blue, respectively, where the switch OFF/ON corresponds to 0/1 logic input conditions. *S*_21_ transmission signal in green corresponds to the logic output and fulfills the AND truth table.

We also implemented 3-bit logic gates by adding a third voltage source *V*_C_ (0.44 V) with series resistor *R*_C_ (50 Ω) and switch C, connected in parallel with the other two voltage sources, *V*_A_ (0.4 V) and *V*_B_ (0.7 V), in the electrical circuit shown in Fig. 1a. Figure 8 shows the frequency responses of the resonator for different logic input conditions with an a.c. actuation voltage of 2 dBm (0.28 *V*_rms_) and *V*_d.c._ of 40 V at 1 torr pressure and at room temperature. Three-bit NOR gate is realized by choosing the a.c.-driving frequency at 119.022 kHz marked in black circle as shown in Fig. 8. For (0,0,0) logic input condition, the frequency response shows high *S*_21_ transmission signal corresponds to the logic output (1). For all the other logic input conditions, the response shows low *S*_21_ transmission signal at this frequency, which corresponds to the logic output (0). Similar to the 2-bit OR logic operation, a 3-bit OR logic function can be realized by selecting the frequency of the anti-resonance dip as the a.c.-driving frequency. Next, a 3-bit AND gate is realized by choosing the frequency of operation at 132.105 kHz marked in black circle in Fig. 8, where only (1,1,1) logic input condition shows high *S*_21_ transmission signal corresponds to the logic output (1). For all the other logic input conditions shows low *S*_21_ transmission signal corresponds to the logic output (0). By selecting the corresponding anti-resonance frequency as the a.c.-driving frequency, a 3-bit NAND gate can be realized. Figure 9a shows the time response of the 3-bit NOR logic gate at the operation frequency of 119.022 kHz. Three logic input signals, A, B and C are shown in black, red and blue, respectively, where the switch OFF/ON corresponds to 0/1 logic input conditions. *S*_21_ transmission signal in green corresponds to the logic output, and fulfills the NOR truth table. Figure 9b shows the demonstration of a 3-bit AND logic function at the a.c.-driving frequency of 132.105 kHz. The output response shown in green fulfills the AND truth table.

One final remark is regarding the chosen d.c. bias voltage of this study. The demonstrated logic gates can be also operated at lower d.c. bias voltage. For example, we demonstrated a 2-bit NOR logic gate with a 20 V d.c. bias voltage (see Supplementary Note 2 and Supplementary Fig. 2a,b).

## Discussion

An important feature of a logic gate is the operation speed. The speed of operation of the proposed logic device is governed by the speed of the electrothermal frequency modulation and the resonator switching speed. The characteristic time associated with electrothermal heating and cooling is typically much longer than the period of free vibrations of MEMS/NEMS structures^26^,^27^. Hence, electrothermal actuators have been mainly explored for static or low-frequency operations^26^,^27^. It is possible to calculate the thermal time constant of the microbeam^28^,^29^ using the equation
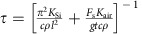
, where *l* is the length of the microbeam, *g* is the gap between the beam and the substrate, *t* is the thickness of the beam, *ρ* is the density of silicon, *c* is the heat capacity of silicon, *K*_Si_ and *K*_air_ are the thermal conductivity of silicon and air, respectively. The beam shape factor, *F*_s_, is a correction term that depends on the geometry of the beam. This correction term is necessary because the heat is conducted to the substrate not only through the bottom surface of the beam but also from the sides and the top surface. The formula for the shape factor^30^ is given by 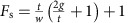, where *w* is the width of the beam. *F*_s_ for the studied microbeam is calculated to be 12.33. The calculated thermal time constant for the microbeam used in this study is 152 μs, which indicates an electrothermal switching speed of 6.5 kHz. The theoretical open-loop switching speed of the MEMS resonator is estimated to be, *f/Q*∼238 Hz. Thus, it can be inferred that the maximum operating speed of the proposed logic device is limited by the ring-up or ring-down time of the resonator rather than the thermal time constant. It is worth to note that by scaling the device dimensions to nanoscale, both the mechanical response time and the thermal time constant will be improved significantly. As an example, we have estimated the thermal time constant^28^,^29^ to be in the order of 10^−6^ s for a clamped–clamped beam resonator with a length of 20 μm, width of 300 nm and thickness of 500nm (ref. 12). This translates into a maximum electrothermal modulation speed in the order of 10^6^ Hz. For the same resonator, the reported open-loop operation speed was around 48 kHz (ref. 12). It implies that the operation speed of logic devices built in these dimensions will be defined by the mechanical response time rather than the thermal response time. By considering a length of 600 μm and width of 50 μm for our device (includes electrodes and anchors), an integration density in the order of 10^4^ per cm^2^ can be achieved. Moreover, we note that the use of nanomechanical resonators would significantly increase the integration density. For a resonator with a length and width of 1 μm and 100 nm (resonance frequency around 1 GHz)^10^, respectively, an integration density as high as 10^8^ devices per cm^2^ is plausible.

Another important aspect of a logic gate is the switching energy necessary to perform the desired logic operation. In this proposed scheme, the energy provided for the necessary switching events for the logic operation is in the form of resistive heating of the microbeam using the electrothermal circuit consisting of *R*_A_, *R*_B_ and *R*_MB_. While only a fraction of the total energy provided to the system is used by the microbeam for the state change during the logic operations, most of the energy is lost in the form of heat dissipation to the environment through *R*_A_, *R*_B_ and *R*_MB_. We estimated the maximum power cost for performing a single-logic operation as 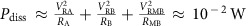. One can note that this energy cost is relatively high compared with other reported energy cost in performing a single-logic operation on nanomechanical resonator-based systems, such as in the work of Guerra *et al*.^12^ and Wenzler *et al*.^21^, which is based on electrostatic actuation. As traditionally well-known, thermal actuation, which is the base of this work, is considered less-energy efficient compared with other actuation methods. Nevertheless, the same principle demonstrated in this work applies when using other actuation techniques, as long as they can actively tune the stiffness of the resonating structure. It is also expected that the energy cost can be further reduced by orders of magnitude by optimizing device geometry.

The sensitivity of the proposed device to temperature variation is another important factor that needs to be addressed. The bandwidth, Δ*f*, of the resonator of this study is estimated to be around 240 Hz (Δ*f=f/Q*) at 1 torr. It implies that for the resonance frequency chosen as the operating frequency, the device will perform the desired logic operation successfully as long as the frequency shift due to the change in the ambient temperature lies within ±120 Hz. We estimated the frequency shift due to temperature change according to ref. 31 *f*(*T*)=*f*_0_(1+*TC*_*f*_(*T*−*T*_0_)), where *TC*_*f*_=−30 p.p.m. per °C, is the temperature coefficient of frequency for silicon resonators^32^. For the ambient temperature change between −10 °C and +60 °C from room temperature at 25 °C, the frequency shift is estimated to be *f*_shift_=±120 Hz, which is within the bandwidth of the resonator. Hence, the device will perform the desired logic operations successfully by selecting the resonance frequency as the driving frequency within this range of temperature variations. Additional temperature compensation scheme would be necessary to perform successful logic operation beyond this temperature range for the current device. Apart from this, the variation of resonance frequency due to phase noise is estimated to be around 105 Hz (see Supplementary Note 3 and Supplementary Fig. 3). Hence the device can still perform the desired logic operation successfully at a given operating conditions since the bandwidth is larger than the noise related frequency shift.

With regards to the potential interference between series and parallel resonances while selecting the a.c. operating frequency, it is noted that lowering down the compensation capacitance will broaden the separation between the series and parallel resonances. Also, improving the bandwidth will help to choose proper operating frequencies with lower margin of error.

A note is worth to be mentioned regarding the survivability of the resonators to mechanical shock. As was demonstrated^33^,^34^,^35^,^36^ theoretically and experimentally, microstructures similar to the studied resonator shows excellent shock resilience up to 30,000–50,000 g. Downscaling the dimensions of the resonators will further improve the shock resilience.

The flexibility to cascade multiple gates is of paramount importance for realizing complex logic circuits. For the proposed scheme it is limited by two current challenges that warrant more future research. First, the strength of the output a.c. signal, which requires a transimpedence amplifier. Second is the fact that the signal waveforms as logic inputs and logic outputs are of different form. The output signal, a.c., needs to be converted into a d.c. signal. The d.c. output signal can be then used as an input to the next logic element, and hence enables sequencing. Also, the d.c. current can be split into various branches or pass through multiple in-series resonators. If a single operating frequency is desired to be used throughout the grid of logic resonators, then one possibility is to fabricate several devices to have slightly different resonance frequencies, such that all can be driven at the same frequency. Also, the devices can be individually tuned by a separate d.c. biasing mechanism for each.

In summary, we demonstrated a reprogrammable logic device based on electrothermal tuning of the resonanance frequency, capable of performing all the fundamental 2-bit logic operations; AND, NAND, OR, NOR, XOR, XNOR and NOT, at room temperature and at modest vacuum conditions. We also demonstrated a single MEMS resonator-based reprogrammable 3-bit AND, NAND, OR and NOR logic gates. This device can be easily modified to perform *n*-bit OR/NOR and AND/NAND logic operations by simply adding one voltage source per bit in parallel in the electrical network responsible for the electrothermal frequency modulation. We program the device to perform a desired logic operation by simply choosing appropriate a.c.-driving frequency. This logic device operates in the linear regime of the resonator, and hence, may further reduce the voltage load if operated under low damping conditions. Although we have used an arch-shaped microbeam resonator, the same principle of electrothermal frequency modulation is equally applicable for a straight clamped–clamped MEMS/NEMS resonator. In fact, the demonstrated principle applies on any MEMS/NEMS resonator devices working in the linear frequency regime with a proper frequency tuning mechanism that can alter the stiffness property of the resonator, and hence, its linear resonance frequency. Future directions in this research can be targeted to simplify the bulky *S*_21_ parameter measurement set-up used in this paper. This complexity can be minimized by integrating necessary complementary metal oxide semiconductor devices, such as transimpedance amplifier, on-chip. This practical demonstration of essential elements of computation using MEMS resonators provide fundamental building blocks for alternative computing scheme in the electromechanical domain.

## Additional information

**How to cite this article**: Hafiz, M. A. A. *et al*. Microelectromechanical reprogrammable logic device. *Nat. Commun.* 7:11137 doi: 10.1038/ncomms11137 (2016).

## Supplementary Material

Supplementary Information Supplementary Figures 1-3 and Supplementary Notes 1-3
