# Supplementary material for: Microelectromechanical reprogrammable logic device
Source: Nat Commun. 2016 Mar 29;7:11137. doi: 10.1038/ncomms11137 (PMC4820632; doi:10.1038/ncomms11137)
Supplement: Supplementary Information — Supplementary Figures 1-3 and Supplementary Notes 1-3 [file ncomms11137-s1.pdf]

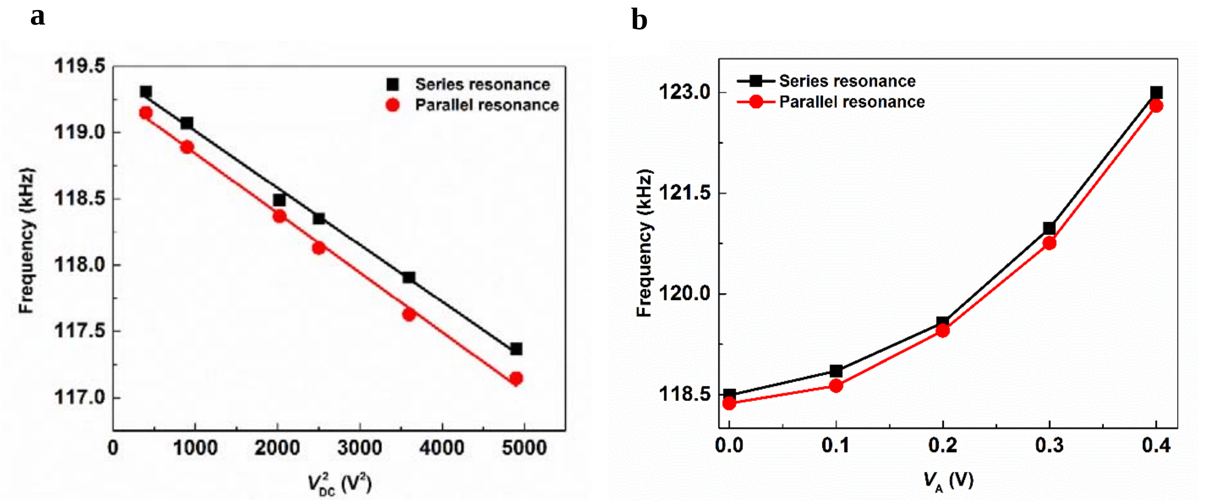

Supplementary Figure 1 | DC bias voltage and electrothermal voltage tuning of the resonance frequency. Variation of the series and the parallel resonance frequency with (**a**) squared of DC bias voltage (V^2_{DC}) (**b**) electrothermal voltage (V_A). The frequency shift due to the elctrothermal voltage is greater than that due to the DC bias voltage.

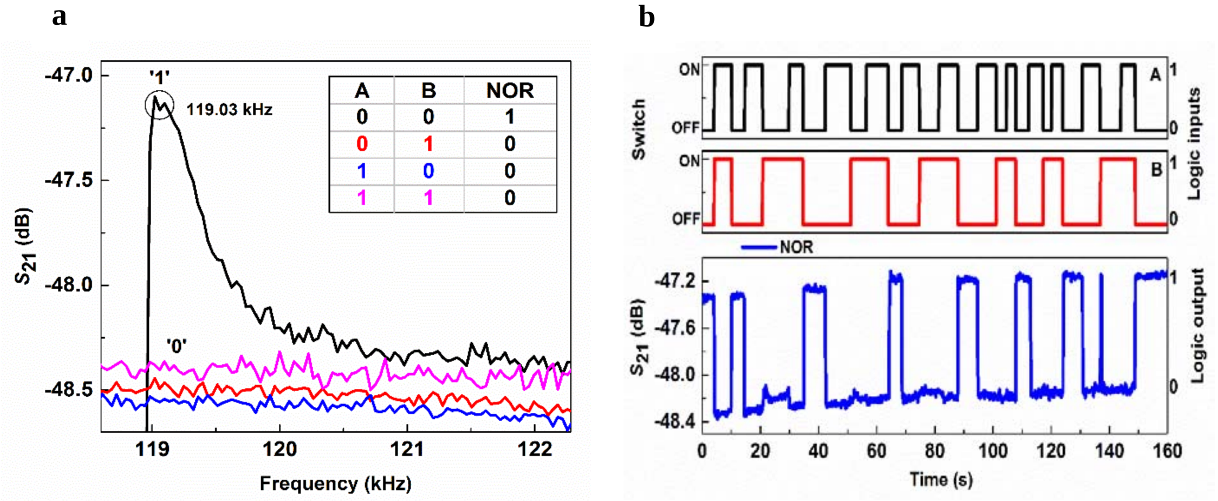

Supplementary Figure 2 | Demonstration of a 2-bit NOR logic gate operation at V_{DC} = 20V and V_{AC} = 2dBm. (**a**) Frequency responses of the resonator for different logic input conditions where (0,0) logic input condition (in black) shows high S_{21} transmission signal (1) at 119.03 kHz and for other logic input conditions shows low S_{21} signal (0). Truth table of the NOR logic gate is shown in the inset. (**b**) Demonstration of NOR logic operation when the frequency of the AC input signal is chosen as 119.03 kHz. Two input signals, A and B are shown in black and red, respectively, where the switch OFF/ON corresponds to the 0/1 logic input conditions. S_{21} transmission signal in blue corresponds to the logic output and fulfills the NOR truth table.

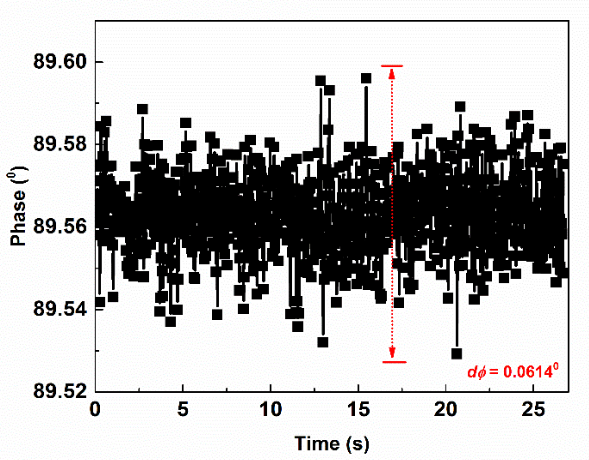

Supplementary Figure 3 | Phase noise analysis. Variation in the phase with time at a constant frequency of 117.663 kHz.

Supplementary Note 1: Dependence of the series and parallel resonance frequency on the DC bias voltage and the electrothermal voltage

Here, we show the characteristics of the resonance frequency for an arch resonator while varying the DC bias voltage and the electrothermal voltage. The variation of the series and parallel resonance frequencies of the resonator with the square of the DC bias voltage is plotted in Supplementary Figure 1a.

Electrothermal frequency modulation has an essential role in the execution of logic functions in this architecture. To see the effect of the electrothermal voltage on the resonance frequency of the resonator, the voltage across the resonator (V_A) is changed by fixing the AC actuation voltage at 2dBm and the DC bias voltage at 45V. The variation in the resonance frequency of the resonator with the electrothermal voltage is shown in Supplementary Figure 1b. For an electrothermal voltage of 0.43V, the frequency of the resonator is increased by 4.51 kHz.

Supplementary Note 2: Logic operation at low DC bias voltage

We have performed an experiment to demonstrate 2-bit NOR logic operation at low DC bias voltage condition on a similar device. Logic operation is performed at a pressure of 1 Torr with an actuation voltage of 2dBm and a DC bias voltage of 20V. Note that all other logic operations can be realized by properly selecting the operation frequencies. Also, by operating at low pressure, one can further reduce the voltage load and can perform all the logic operations by selectively choosing the AC driving frequency. Supplementary Figure 2a shows the operation of a 2-bit NOR logic gate at V_{DC} = 20V and V_{AC} =2dBm. Execution of the NOR logic is demonstrated in Supplementary Figure 2b.

Supplementary Note 3: The effect of phase noise on the device performance

In order to understand the effect of phase noise at a given temperature on the performance of the device, we estimated the frequency fluctuations due to phase noise of the resonator. The shift in the resonance frequency can be obtained through the change of phase signal measured at a constant excitation frequency. From the measured frequency response of the electrical signal, the phase slope of the linear regime around resonance is determined to be $d\phi/df$ = 5.84457 x 10^{-4} per Hz. A study of the phase evolution at a fixed frequency of 117.663 kHz has been performed, which is shown in the Supplementary Figure 3. Accordingly, the phase noise is determined to be $d\phi_{\min}$ =0.0614^0, which implies a frequency shift given by $df = \frac{d\phi}{d\phi/df}$ =105 Hz. It is seen that the frequency fluctuation of the resonator lies within the bandwidth of the resonator. Hence, the defined logic state would still remain intact at a set operating frequency and the device would perform the desired logic operation successfully.
